# Editorial

**Published:** 1913-01

**Authors:** 


### EDITORIAL

The Eusiness Office of the *Journal-Record* is 23 West Alabama Street.

The Editorial Office is 1014-15 Atlanta National Bank Building.

Address all Business Communications to *Journal-Record of Medicine*, 23 West Alabama Street.

Make remittances payable to *The Journal-Record of Medicine.*

On matters pertaining to the Editorial and Original Communications, address Edgar G. Ballenger, M. D., Atlanta, Ga.

Reprints of Original Articles will be furnished at cost price. Requests for the same should always be made in THE MANUSCRIPT.

We will present, postpaid, on request to each contributor of an original article, twenty (20) marked copies of *The Journal-Record of Medicine* containing such article.

### THE COMPARATIVE FREQUENCY OF GONORRHOEA IN THE WHITE AND COLORED RACES

Elsewhere in this number we publish a letter from Dr. Wm. A. Steele, of Philadelphia, requesting first hand information regarding the relative frequency and results of gonorrhoea in the white and colored races. It is, we believe, the current opinion in the South, where the negro population is so large, that gonorrhoea is both less frequent and less severe in the negro than in the white race. Were it as common and difficult to cure in the negro the inadequate treatment the majority of them secure, would result in effects and complications being much more prevalent and serious. Their somewhat lax morals would also cause a rapid diffusion of the disease, and its frequency would not be a question in doubt. That they are in a way more or less immunized to syphilis, we also believe to be true. The rarety and infrequency of serious luetic affections and late nervous manifestations seem to indicate this fact. Almost never, do they take an adequate course of antisyphilitic treatment. That the male negro is nearly always infected with gonorrhoea, as Dr. Taylor states is, we believe, untrue. If it were the ravages of this disease it would be only too apparent. We are quite willing to admit that chronic prostatic inflammation plus alcoholic irritation would stimulate sexual desire and remove the normal restraining influence. That this is the cause of assaults upon white women certainly is not proven nor to us does it seem the prime factor. It is not, to our knolwedge, a common thing for the assaulted woman to develop gonorrhoea. We would be glad to have any physician who reads this, to inform us if he personally knows of any such infecton. Gonorrhoea we think, is more prevalent in the city than country negroes. The proportion of gonorrhoeal ophthalmia is greater among the negroes than white, probably because so few of them have trained physicians to apply credes prophylactic measures.

We will be glad to have our readers, who are interested in this subject, answer the questions and give their opinions to Dr. Steele.

---

### SOCIALISM AND THE MEDICAL PROFESSION

The most gigantic experiment in medical socialism which the world has ever seen is about to be undertaken in England and its results will be watched with the greatest interest by philanthropists, socialists and particularly by the physicians the world over. Similar plans have been tried in other countries with more or less success but the British National Insurance Act which has just gone into effect in England includes many new features and is vastly more far-reaching than anything of the kind which has ever been tried. The number of insured persons will be more than 13,000,000 and will include all those, both men and women, whose yearly wage is less than $800. Eight cents a week will be deducted from the wages of all men and six cents from the wages of women and the balance of the fund, which will amount to more than $50,000,000 a year will be contributed partly by the employers and partly by the government. The benefits to be received will be $2.50 per week for men and $1.85 per week for women during twenty-six weeks of illness and $1.25 per week for both men and women, when they are ill longer than twenty-six weeks. Free medical attendance and free medicines are also included and there is a material benefit of $7.50 at the birth of every child. In addition there is free sanitarium treatment for consumptives. Of course the success or failure of this vast scheme of contract medical practice will depend upon the attitude of the medical profession towards it, and that attitude at the present time is distinctly hostile. It is stated that more than

27,000 members of the British Medical Association have declined to serve under the act, which practically nullifies it for the time being. The reason for this is that the compensation offered is so pitifully small that it simply means a tremendous amount of work for the physicians who might be employed, at a compensation which no one could afford to work for. In other words, while the government would get the credit for an enormous philanthropic work, the real charity would come out of the pockets of the medical profession. Every physician the world over, does and is glad to do a great deal of work for the sick poor for nothing, but he likes to do it spontaneously and not have it forced upon him. Under the British insurance plan the patient would have the right to demand the most exacting service, in return for the very small amount he pays in his weekly dues, and the physician would receive in return for a vast amount of work a very trifling compensation. The injustice of this demand upon the medical profession of England, many of whom under present conditions are almost starving under the yoke of various forms of contract practice, is recognized by many thoughtful persons and there will no doubt be brought about a readjustment of the scale of fees as at present contemplated by this act, which will enable the medical profession to accept positions under it. We are inclined to think that the time will come when the relations of the medical profession and the people will be greatly changed and the physician will be employed by the state rather than by the individual. In the public support of Preventive Medicine and in the employment of health officers the state recognizes now a duty to the people in maintaining so far as possible healthy and sanitary conditions and protecting its citizens from disease; it is but a short step forward to recognize a duty to cure its citizens when afflicted. As the chief asset of every community is to be found in the lives of its healthy citizens, the economy of taking care of the health of its citizens becomes obvious. Such plans as are contemplated by the National Insurance Act of Great Britain, vicious though that act seems to be in some of its details, show a distinct tendency towards a form of socialism which in its ultimate development will undoubtedly make for human welfare and human happiness.—*Editorial St.Paul Medical Journal, January* 1913.

### BETTER HOSPITAL FACILITIES NEEDED

Unfortunately there are no hospital facilities for the treatment of acute gonorrhoea or syphilis in Atlanta. The excuse given is that both space and funds are limited. Such ailments as these which are often considered trivial, must remain uncured until they reach a stage where a serious abdominal operation or some other grave malady arises then the hospital opens its doors to them, but not until they have spread the disease to others, perhaps innocent women and children. Statistics show that more than 75% of abdominal operations are the result of gonorrhoea. Would it not be a matter of economy to cure this disease early and thus cut down the surgical work, and let the space and money be devoted to the diseases that cause so many operations, and so much expense? The same line of reasoning applies to syphilis, the chief cause of insanity, locomoter ataxia, aneurysm and many other serious ailments that at a late stage of the disease crowd the hospitals.

Are we not grossly inconsistent to decline to provide treatment for these diseases early? Our wards to-day are probably crowded with patients who could much more easily have been cured years ago before the serious after-effects have arisen and before the disease had been spread to many others who continue to transmit it and grow worse waiting for something serious to happen so that the city hospital can care for them.

Minneapolis carried out an active campaign of suppression about two years ago and in order to determine if this had resulted in any good, we directed letters to the mayor and about fifty citizens asking their personal opinion as to the actual results that followed these measures. As this city is about the size of Atlanta, we thought the plan carried out there might also work successfully here. The concensus of opinion however, as expressed in the replies to our letter, cleearly indicate that the condition had at least been little if any improved, while many insisted that it was worse than before suppressive measures were adopted. Many of these in favor of suppression admitted that in Minneapolis it could not be considered successful as the scattered prostitutes had been brought in contact with a larger number of citizens than when they were restricted to a certain area, and that the demoralizing effect was greater. The letters from physicians also indicated that there had been a perceptible increase in gonorrhoea and syphilis. Those among us favoring suppression have in the past held Minneapolis up as a notable example of the success of suppressive measures.

